# Enhanced Static Modulated Fourier Transform Spectrometer for Fast Approximation in Field Application

**DOI:** 10.3390/molecules26113312

**Published:** 2021-05-31

**Authors:** Ju Yong Cho, Won Chun Oh, Won Kweon Jang

**Affiliations:** 1 Department of Aeronautic Electricity, Hanseo University, Seosan 31962, Korea; food0421@naver.com; 2 Department of Advanced Materials Science & Engineering, Hanseo University, Seosan 31962, Korea

**Keywords:** InGaAs, light emitting diode, energy band gap, static modulation, spectrometer

## Abstract

We discuss the data sampling frequency, the spectral resolution, and the limit for non-aliasing in the static modulated Fourier transform spectrometer based on a modified Sagnac interferometer. The measurement was performed in a very short 4 ms, which is applicable for real time field operation. The improved spectrometer characteristics were used to investigate the spectral properties of an InGaAs light emitting diode. In addition, The measured spectral peak was shifted from 6420 cm^−1^ to 6365 cm^−1^, as the temperature increased from 25 °C to 40 °C, when the operating current is fixed to be 0.55 A. As the applied current increased from 0.30 A to 0.55 A at room temperature, the spectral width was broadened from 316 cm^−1^ to 384 cm^−1^. Compared to the conventional Fourier transform spectrometer, the measured spectral width by the static modulated Fourier transform spectrometer showed a deviation less than 10%, and the spectral peak shift according to the temperature rise showed a difference within 2%.

## 1. Introduction

Fourier transform infrared spectroscopy has been widely used in analyzing the properties of materials because it is possible to collect spectral information in a very wide wavelength range in a relatively short time. However, the measurement has usually been performed in a laboratory environment due to the strict operating conditions. Recently, the research for the static modulated Fourier transform spectrometer of various structures has been reported, because it can provide the fast and outdoor measurement with remote sensing operation. However, it is still suffering from relatively low spectral resolution and data reliability. Nevertheless, the number of cases in which measurements must be made remotely in a very short time from outdoors are increasing, and various studies have been reported as countermeasures. In fields of health and medical application, the techniques for diagnosing diseases or determining material properties are often being introduced using spectrometry, and the employed systems are usually big and complicated, because most spectroscopic systems are based on diffraction distribution. However, the systems of diffraction distribution need a long measurement time to compensate the weak intensity of diffracted signal that pass through the slit. Additionally, it also has a drawback in spectral resolution when it measures the optical properties of material in the infrared range, because the diffraction angle is becoming smaller as the wavelength is becoming longer [1,2]. On the other hand, the spectral resolution of a Fourier transform spectrometer is being better as the wavelength is being longer. Therefore, the Fourier transform spectrometer is a better choice in finding out the spectroscopic properties of materials with absorption peaks in the infrared region, because many harmful or toxic materials have their spectral absorption peaks in the infrared region. 

In spite of these many advantages, the conventional Fourier transform spectrometers have been limited by some intrinsic drawbacks. A mirror in the system must be precisely moved under the strictly controlled operation environment to isolate any mechanical instability, to get an interferogram as a function of time. Therefore, the conventional Fourier transform spectrometers are usually being operated in the laboratory and the samples have to be brought from the site to the laboratory to place it inside of the instrument. It is fatal for the materials of fast changing property and the misanalysed results can lead to the unintended solution or delay the development of the solution. 

In order to overcome these disadvantages, much research on the mechanically stable and fast measurement Fourier transform spectrometers has been reported [3,4,5,6,7,8]. Although most of them could solve the problems of the conventional Fourier transform spectrometer, a recent apparent problem of the comparatively low spectral resolution has come to light. It is caused by the limitation of the obtainable maximum optical path difference and insufficient data sampling frequency. To solve these problems, several methods have been suggested, such as the sensor shift and warping compensation using a two-dimensional image sensor [3,4,5], imaging polarization based on Wollaston prisms [6], stepped mirror [7] and birefringent retarder array [8]. However, these attempts were not enough to solve the problem of low spectral resolution, and the structural parameters of refractive optics became another obstacle of the intensity reduction of incident light. Although there are many advantages of spatial modulation, the spectral resolution can be limited by the insufficient number of sampling data and the maximum optical path difference. The physically obtainable number of sampling data is determined by the number of pixels of the detector, and the maximum optical path difference is limited by the incident beam and the optical system size.

In this paper, we suggest the static modulated Fourier transform spectrometer based on the modified Sagnac interferometer features having higher optical throughput and longer optical path difference compared to other static modulated Fourier transform spectrometers. Higher optical throughput was possible because there are no absorptive or refractive optics, and longer optical path difference could be implemented due to the modified Sagnac interferometer. Nonetheless, there were two shortcomings of the insufficient number of sampling data and the limitation for the obtainable maximum optical path difference, because the generated interferogram appears in the overlapped region of two beams that traveled along different optical paths in the interferometer. In order to overcome these shortcomings, we proposed the signal padding method that has already been introduced in our former work [9]. The signal padding method that we used, provides the concept that zero value can be inserted to the both ends of the interferogram to extend the maximum optical path difference, assuming that the padding signal as a real signal at high optical path difference [9]. In our former study, Relationship between the number of sampling points in the overlapped region and the spectral resolution was mainly discussed, and it was confirmed that the spectral distortion due to low spectral resolution can be recovered by the signal padding method, whereas in this study, we discussed the change of data sampling frequency according to mirror displacement, and the relationship between the data sampling frequency and the incident light frequency is also discussed to find out the condition for best spectral resolution without the aliasing. In the spatial modulation, the data sampling frequency and the maximum optical path difference are related to the spectral distortion. In the smaller maximum optical path difference, the data sampling frequency becomes relatively high. However, the spectral information can be incorrect due to the lower spectral resolution caused by the small maximum optical path difference. Though the signal padding method can be used to improve the spectral resolution, it becomes less effective. In the larger maximum optical path difference, the data sampling frequency does not become high enough. In this case, the spectral information can be distorted by aliasing. In addition, the aliasing in the spectral information is related to not only the data sampling frequency, but the incident light frequency, and the measurement for wide spectral light sources is required in the field applications.

Especially, in the field applications, the measurement for wide spectral light sources should be performed, which means that the condition for maximizing spectral resolution without the aliasing is important. Furthermore, the measurement time is shortened from several minutes to 4 ms compared to the conventional Fourier transform spectrometers, and it is much shorter than the 20 ms that was reported in our previous work [9]. The mechanical stability is improved by changing the data acquisition from time function to spatial function, and the system compactness is possible by removing a moving mirror in the system.

In this study, the spectrum of InGaAs light emitting diode was measured using the static modulated Fourier transform spectrometer based on the modified Sagnac interferometer. The changes in the spectral peak and spectral width according to applied current and temperature values were investigated in theory and experiment. Experimental results by the static modulated Fourier transform spectrometer are compared with the results measured with a diffraction-based spectroscope to find out how accurate the results can be obtained when it is operated in the field.

## 2. Measurement Methods

### 2.1. A Static Modulated Spectrometer Based on a Modified Sagnac Interferometer

**Figure 1:**
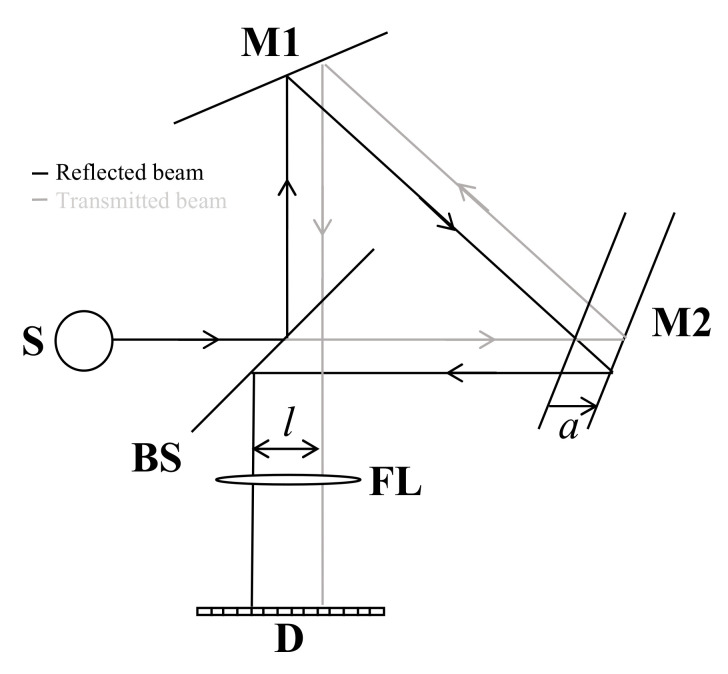
Scheme of the static modulated Fourier transform spectrometer based on a modified Sagnac interferometer. S is a source, M1 is a fixed mirror, BS is a beam splitter, FL is a focusing lens, D is a one-dimensional detector, and M2 is a displaced mirror. From the BS, black and grey solid lines indicate the reflected and the transmitted beams, respectively.

The modified Sagnac interferometer structure consists of a beam splitter and two fixed mirrors. The beam splitter is tilted by 45° about the optical axis, and the two mirrors are inclined by 25° about each incident surface. Figure 1 shows the modified Sagnac interferometer structure. After the incident beam is divided at the beam splitter as reflected and transmitted beams, the distances between beam splitter to each mirror should be asymmetrical to have an optical path difference. When M2 is shifted as much as a from the symmetrical position, the transmitted and reflected beams proceed apart by the distance l, and it can be expressed as Equation (1).

(1) $$l=\sqrt{2}a$$

where a is the displacement of a mirror M2 from symmetrical position, and l is the distance between two separated beams.

**Figure 2:**
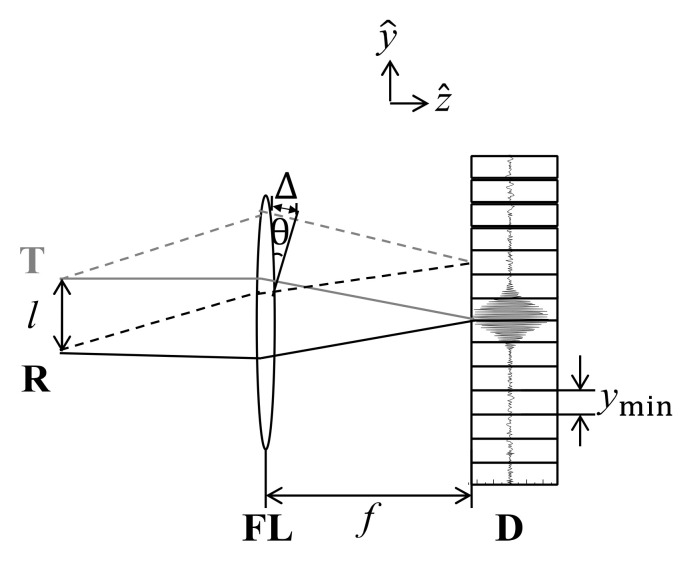
Schematic view of interferogram detection. T and R are transmitted and reflected beams, FL is a focusing lens and D is the one-dimensional detector.

The optical path difference ∆ at the position in the focal plane can be regarded as the same as the optical path difference occurring in the double slit, as shown in Figure 2. Therefore, ∆ can be expressed as Equation (2).

(2) $$∆=l\cdot \sin\theta \approx l\cdot \theta =l\frac{y}{f}=l\frac{n\cdot y_{\min}}{f}$$

where ∆ is the optical path difference, y is the position at the focal plane, n is an integer indicating pixel position, and $y_{\min}$ is a pixel pitch. Moreover, θ is the subtended angle.

In the static modulated Fourier transform spectrometer, the spectral resolution is dependent of the optical path difference. Larger optical path difference is needed to obtain a better spectral resolution. Additionally, the wavenumber ${\bar{\nu }}_{p}$ is related to the optical path difference between adjacent pixels of the detector, which can be expressed as Equation (3).

(3) $${\bar{\nu }}_{p}=\frac{f}{l\cdot y_{\min}\cdot n}$$

Spectral information can be distorted due to aliasing. In order to prevent the distortion, the data sampling frequency of the static modulated Fourier transform spectrometer ought to satisfy the Shannon criterion. The data sampling frequency should satisfy the following condition.

(4) $$\frac{{\bar{\nu }}_{\mathrm{Sample}}}{{\bar{\nu }}_{\mathrm{Source}}}=\frac{f/\left(l\cdot y_{\min}\right)}{{\bar{\nu }}_{\mathrm{Source}}}\geq 2$$

where ${\bar{\nu }}_{\mathrm{Source}}$ is the frequency of source and ${\bar{\nu }}_{\mathrm{Sample}}$ is the data sampling frequency of the static modulated Fourier transform spectrometer comprised of the modified Sagnac interferometer. In the low spectral resolution, the data sampling frequency becomes high due to small values of l and f. In this case, though the spectrometer satisfies the Shannon criterion, the spectral information can be inprecise due to a low spectral resolution. Therefore, the most proper value of the ratio in Equation (4) for the best obtainable spectral resolution is about 2. However, if l is long, even when the ratio in Equation (4) is 2, the overlapped sector becomes small, which results in insufficient data sampling points to express the spectral information. In this case, the signal padding method can be effective to overcome the problem.

### 2.2. Bandgap Change According to Temperature Variation in a InGaAs Based Semiconductor

In semiconductors, the energy band gap depends on the lattice constant that changes with temperature and the interaction between electrons and lattice. The temperature dependent energy band gap can be expressed as Equation (5) in the case of InGaAs [10,11,12].

(5) $$E_{g}\left(T\right)=E_{g}\left(0\right)-\frac{\alpha T^{2}}{\beta +T}$$

where $E_{g}\left(T\right)$ is the energy band gap at temperature T, $E_{g}\left(0\right)$ is the energy band gap at 0 K, and α and β are Varshini’s fitting parameters [7]. β is proportional to the Debye temperature [13].

The center wave number ${\bar{\nu }}_{\mathrm{center}}\left(T\right)$ and the energy band gap $E_{g}\left(T\right)$ can be expressed as Equation (6).

(6) $$E_{g}\left(T\right)=\frac{hc}{\lambda_{\mathrm{center}}\left(T\right)}=\frac{1.24}{\lambda_{\mathrm{center}}\left(T\right)}=\frac{1.24∆{\bar{\nu }}_{\mathrm{center}}\left(T\right)}{10^{4}}$$

where h is Planck’s constant, c is the speed of light, $\lambda_{\mathrm{center}}\left(T\right)$ is the center wavelength in µm at temperature T, and ${\bar{\nu }}_{\mathrm{center}}\left(T\right)$ is the center wavenumber at temperature T.

### 2.3. Experimental Setup

The inspected light source was InGaAs light emitting diode with a center wavenumber of 6451 cm^−1^, a spectral width of 370 cm^−1^ and an output power of 2 mW. The emission spectrum was measured while changing the temperature and current. In order to measure the temperature-dependent energy band gap change, the applied current was fixed at 0.55 A and the temperature increased from 25 °C to 40 °C, and to measure the current dependent change, the temperature of the device was fixed at 25 °C and the current increased from 0.30 A to 0.55 A.

In the modified Sagnac interferometer structure, M1 and M2 are silver-coated square mirrors with a width of 2 inches. The beam splitter has 50% transmittance at 45° in the wavelength range of 3921~11,111 cm^−1^, and 45° incline for the optical axis. M1 and M2 are inclined by 22.5° for the each incident surface. M2 shifted 3.9 mm from the symmetrical position to create the optical path difference, which was determined in consideration of the spectral width of the InGaAs light emitting diode and the temperature dependent energy band gap change. As for the detector, a linear array detector with a measurement range of 4000 cm^−1^ to 11,111 cm^−1^ and 512 effective pixels was used. Therefore, the measurable wavenumber range is the same as that of the detector. The effective detector length was 12.8 mm, and the width per pixel is 25 μm.

## 3. Results and Discussion

**Figure 3:**
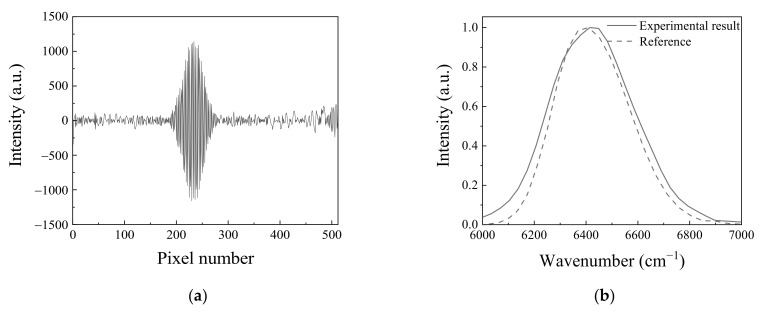
(**a**) The interferogram measured in the static modulated spectrometer with the modified Sagnac interferometer: (**b**) Spectrum comparison. Experimental result shows the spectrum that interferogram in Figure 3a is Fourier transformed and reference shows the spectrum measured using the monochromator. The applied current and temperature to the light emitting diode were 0.55 A and 25 °C, respectively.

In order to evaluate the accuracy of the obtained spectrum from the static modulated interferometer, it was compared with the spectrum obtained using the monochromator. The spectrum was measured under the applied current of 0.55 A and a temperature of 25 °C. Figure 3a shows an obtained interferogram using a static modulated interferometer. Figure 3b shows the comparison of a Fourier-transformed interferogram that is depicted in Figure 3a with a spectrum measured by a monochromator. The spectral resolution and bandwidth of the Fourier transformed spectrum were 34 cm^−1^ and 384 cm^−1^, respectively. It was a little broader than the spectral width of 339 cm^−1^ that was measured by a monochromator. The difference came from the Blackman–Harris apodization function that applied to reduce the sidelobes of the obtained spectrum by the static modulated spectrometer. The difference in the spectral width is about 10 nm, which corresponds to an error within 10%. In this experiment, with the consideration of central wavenumber of the source, theoretical spectral resolution is 26 cm^−1^. However, the spectral resolution was not acquirable because the spectral information was distorted due to aliasing. This phenomenon is from the discrepancy of the spectral peak of the light source. The spectral resolution without the aliasing was 34 cm^−1^. In this case, the calculated ratio using Equation (4) was 2.5.

**Figure 4:**
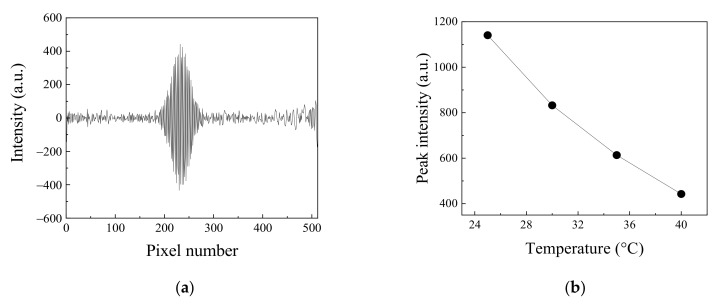
(**a**) The interferogram measured in the static modulated spectrometer with the modified Sagnac interferometer when applied current and temperature were 0.55 A and 40 °C, respectively: (**b**) Peak intensity change in the interferogram according to the change of the temperature of the light emitting diode. Applied current to light emitting diode was fixed to 0.55 A.

The static modulated interferogram was measured to evaluate the temperature-dependent energy band gap change. The measurement was performed when the applied current was fixed to 0.55 A; the temperature increased from 25 °C to 40 °C. As the temperature increased, the emitting spectral intensity of the light emitting diode decreased. Figure 4a shows the measured interferogram when the temperature is 40 °C. In this case, the peak intensity of the interferogram decreased by 61% compared to the maximum peak of the interferogram measured when the temperature of the light emitting diode was 25 °C, as shown in Figure 3a. Figure 4b shows the decrease of peak intensity of the interferogram according to the light emitting diode temperature increase.

**Figure 5:**
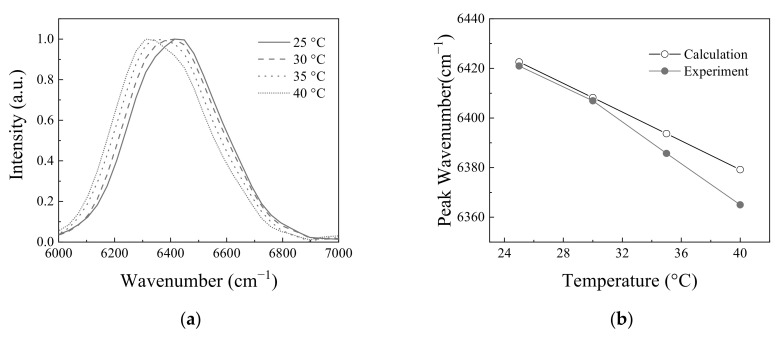
(**a**) Spectrum change according to the temperature: (**b**) The change of peak wavenumber according to temperature. Calculation values are calculated by Equations (5) and (6). Experimental values are from the measured values by static modulated Fourier transform spectrometer. Applied current to light emitting diode was fixed to 0.55 A.

The change of the energy band gap was inspected according to the temperature variation by measuring the interferograms. Figure 5a shows the normalized Fourier transform spectra of measured interferograms. The center wavenumber was 6420 cm^−1^ at 25 °C, and shifted to 6364 cm^−1^ at 40 °C, because the energy band gap becomes smaller as the temperature increases. The temperature-dependent energy bandgap can be calculated with Equation (6). Figure 5b shows the comparison of measured and calculated peak wavenumbers according to the temperature. In calculation, α and β were 4.9 × 10^−4^ eV/K and 327 K, respectively [12]. When the temperatures of InGaAs light emitting diode were 25 °C and 30 °C, the peak wavenumbers measured by the static modulated Fourier transform spectrometer were 6423 cm^−1^ and 6410 cm^−1^, respectively, which almost coincides with the calculated and measured values of monochromator. However, as the temperature increased to 35 °C and 40 °C, the discrepancy became larger between the measured and calculated values. This phenomenon might come from the spectral distortion caused by the intensity-decreased interferogram as the temperature increases.

**Figure 6:**
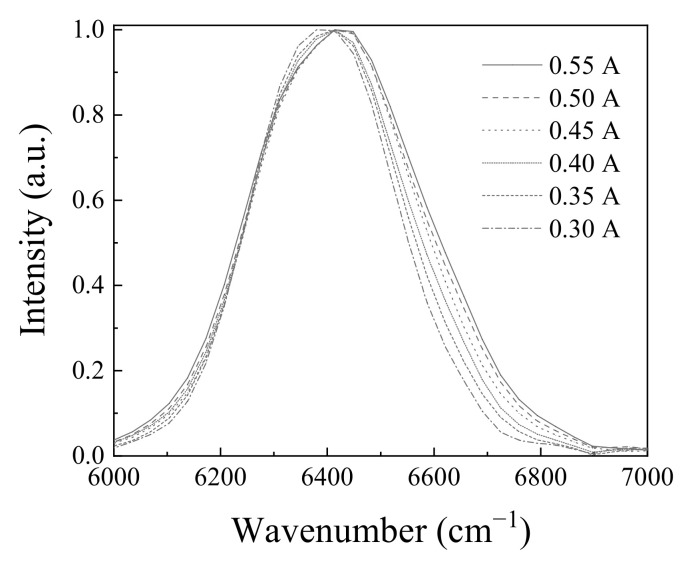
Spectral change of the light emitting diode according to the applied current at room temperature.

Figure 6 shows the spectral change, while the applied current was changed from 0.30 A to 0.55 A. The spectral width widens as the current increases. The spectral width at 0.30 A was measured to be 316 cm^−1^, but it was widened to 384 cm^−1^ at 0.55 A. The spectral widening may be caused by the spectral gain property of the light emitting diode material [13].

## 4. Conclusions

The fast and remote sensing spectral measurement of the InGaAs light emitting diode according to temperature and current was performed by the enhanced static modulated Fourier transform spectrometer based on the modified Sagnac interferometer. In the temperature-dependent energy band gap analysis, the spectral resolution of the static modulated Fourier transform spectrometer was 34 cm^−1^ and the spectral width was 384 cm^−1^, of which the difference was within 10% compared to the theoretical and the monochromator measured values. In the temperature-dependent peak wavenumber shift analysis and applied current dependent analysis, the measured spectral peaks of the static modulated Fourier transform spectrometer showed a coincidence with the theoretical and the monochromator measured values, except higher temperature conditions. Considering the short measurement time of 4 ms in the enhanced spectrometer, it had excellent performance compared to the conventional dynamic modulated Fourier transform spectrometer. Though the static modulated Fourier transform spectrometer based on the modified Sagnac interferometer, proposed in this paper, has a limitation in spectral resolution due to the physically limited number of detector pixels, it provided acceptable spectral information. In this study, we showed the spectral performance of the static modulated Fourier transform spectrometer based on the modified Sagnac interferometer. Due to several advantages such as fast measurement, seismic resistance and compact size, it is possible to be applied for the outdoor operation as a mobile instrument. It is expected to be used for environment surveillance, infection detection, and countermeasure for hazardous gas leakage accidents.

## Data Availability

Not Applicable.
